# Strong Start Innovation: Equitable Outcomes Across Public and Privately Insured Clients Receiving Birth Center Care

**DOI:** 10.1111/jmwh.13439

**Published:** 2022-12-08

**Authors:** Diana Jolles, Lauren Hoehn‐Velasco, Lisa Ross, Susan Stapleton, Jennie Joseph, Jill Alliman, Kate Bauer, Ebony Marcelle, Jennifer Wright

**Affiliations:** ^1^ Frontier Nursing University Versailles Kentucky; ^2^ Department of Economics Georgia State University Atlanta Georgia; ^3^ American Association of Birth Centers Perkiomenville Pennsylvania; ^4^ Commonsense Childbirth Winter Garden Florida; ^5^ Midwifery Melanated, LLC Washington District of Columbia

**Keywords:** antepartum care, birth centers, health equity, vulnerable populations

## Abstract

**Introduction:**

The Birth Center model of care is a health care delivery innovation in its fourth decade of demonstration across the United States. The purpose of this research was to evaluate the model's potential for decreasing poverty‐related health disparities among childbearing families.

**Methods:**

Between 2013 and 2017, 26,259 childbearing people received care within the 45 Center for Medicare and Medicaid Innovation Strong Start birth center sites. Secondary analysis of the prospective American Association of Birth Centers Perinatal Data Registry was conducted. Descriptive statistics described sociobehavioral, medical risk factors, and core clinical outcomes to inform the logistic regression model. Privately insured consumers were independently compared with 2 subgroups of Medicaid beneficiaries: Strong Start enrollees (midwifery‐led care with peer counselors) and non‐Strong Start Medicaid beneficiaries (midwifery‐led care without peer counselors).

**Results:**

After controlling for medical risk factors, Strong Start Medicaid beneficiaries achieved similar outcomes to privately insured consumers with no significant differences in maternal or newborn outcomes between groups. Perinatal outcomes included induction of labor (adjusted odds ratio [aOR], 0.86; 95% CI 0.61‐1.13), epidural analgesia use (aOR, 1.00; 95% CI, 0.68‐1.48), cesarean birth (aOR, 1.16; 95% CI, 0.87‐1.53), exclusive breastfeeding on discharge (aOR, 1.11; 95% CI, 0.48‐2.56), low Apgar score at 5 minutes (aOR, 1.23; 95% CI, 0.86‐1.83), low birth weight (aOR, 1.12; 95% CI, 0.77‐1.64), and antepartum transfer of care after the first prenatal appointment (aOR, 1.53; 95% CI, 0.97‐2.40). Medicaid beneficiaries who were not enrolled in the Strong Start midwifery‐led, peer counselor program demonstrated similar results except for having higher epidural analgesia use (aOR, 1.30; 95% CI, 1.10‐1.53) and significantly lower exclusive breastfeeding on discharge (aOR, 0.57; 95% CI, 0.40‐0.81) than their privately insured counterparts.

**Discussion:**

The midwifery‐led birth center model of care complemented by peer counselors demonstrated a pathway to achieve health equity.

## INTRODUCTION

The Strong Start for Mothers and Newborns Initiative (Strong Start), a joint effort between the Centers for Medicare & Medicaid Services, the Health Resources and Services Administration, and the Administration on Children and Families, tested enhanced approaches to prenatal care for beneficiaries of Medicaid.^1^ Birth centers that participated in Strong Start addressed the increased risk for poor perinatal outcomes among their clients who used Medicaid by integrating peer counselors into standard midwifery‐led birth center care. Peer counselors functioned in a variety of capacities including community health workers, doulas, and care coordinators. Using birth certificate data, Medicaid claims data, and propensity score reweighting techniques, the Strong Start impact analysis compared midwifery‐led birth center‐based care to typical care by region and demonstrated that the model of birth center care resulted in decreased rates of preterm birth, low birthweight, and cesarean birth.^1^

Continuing education (CE) is available for this article. To obtain CE online, please visit http://www.jmwhce.org. A CE form that includes the test questions is available in the print edition of this issue.

Birth center care has been recognized as a driver of the *triple aim* for improving population health as it improves quality of care, family experience of care, and value.^1^, ^2^ The Strong Start birth center intervention evaluated midwifery‐led care, which is categorized by the Hallmarks of Midwifery^3^ as a relationship‐based wellness model of prenatal, intrapartum, and postpartum care led by midwives practicing within the continuum of an interprofessional care system.^4^ Consumer engagement, shared decision‐making, and the wellness‐based model of midwifery‐led care were demonstrated to be effective and high value within the Strong Start impact analysis.^1^

Results of the Strong Start impact analysis demonstrated that outcomes depended on the type of prenatal care received (midwifery‐led birth center or typical medical model) rather than the place of birth.^1^ Childbearing people who received prenatal care at a birth center and gave birth in a hospital, either for medical indications or for personal preferences, had better outcomes than the group that had typical medical model prenatal care. The birth center group had more weekend births, demonstrating adherence to the hallmarks of midwifery that promote nonintervention in the absence of complication.^3^ Additionally, outcomes of being cared for within the midwifery‐led system demonstrated impacts to newborns and infants through one year of life, including decreased emergency room visits.^1^

Previous research has demonstrated that pregnant people who are recipients of Medicaid are at higher risk for poor outcomes than those with private health insurance.^5^, ^6^, ^7^ Medicaid eligibility criteria align with the social determinants of health and include lack of financial resources, availability of transportation, housing instability, lack of social support, stress, limited availability of healthy and nutritious foods, lower levels of education, and lack of access to health care that correlate with poor pregnancy outcomes. Medicaid is a marker for the intersectional elements of poverty, which are known to drive poor perinatal outcomes.^1^, ^5^, ^6^, ^7^ Strong Start birth centers demonstrated a mitigating effect on health disparities, narrowing the preterm birth gap for Black and Hispanic birthing people, compared with the matched Medicaid comparison group in the Strong Start impact analysis.^1^, ^8^ The purpose of this research was to advance the state of the science by evaluating the model's potential for decreasing poverty‐related health disparities by exploring the private and public payer outcomes within the 45 Center for Medicare and Medicaid Innovation (CMMI) Strong Start Birth Center Sites.

## METHODS

This is a secondary analysis of the American Association of Birth Centers (AABC) Perinatal Data Registry (PDR). The registry is one of the largest prospective data registries in the United States and is designed for quality assurance, quality improvement, and research of the midwifery‐led birth center model of care. All 10 public health regions of the United States were represented within the sample. The AABC PDR measures more than 900 variables throughout the perinatal episode of care, beginning with the first prenatal visit and stopping at 6 weeks postpartum. All childbearing people beginning care at an AABC PDR user sites were tracked through their disposition including antenatal, intrapartum, and postpartum transfers to higher levels of care if needed.

**QUICK POINTS**

✦ After controlling for medical risks and adjusting for social factors, birth center care resulted in similar perinatal outcomes for Strong Start Medicaid beneficiaries and privately insured populations.
✦ Strong Start Medicaid beneficiaries who received midwifery‐led care complemented by peer counselors showed no significant differences in core clinical outcomes when compared with privately insured beneficiaries.
✦ Non‐Strong Start Medicaid beneficiaries demonstrated similar health outcomes compared with privately insured consumers, except for higher epidural analgesia use and lower rates of exclusive breastfeeding on discharge.

Variables include sociodemographic descriptors, process, and outcome measures and were harmonized with nationally endorsed perinatal care measures as well as with the reVITAlize Obstetrics Data Definitions.^9^, ^10^ The registry adheres to guidelines from the Agency for Healthcare Research and Quality including quality insurance mechanisms to ensure completion of data, systematic patient enrollment, and data consistency checks with medical records.^11^ The AABC PDR has been demonstrated to be reliable and valid, exceeding birth certificate capabilities with 100% consistency for 10 variables when cross‐matched with 2 data sources.^12^ Childbearing families sign written consent to participate upon entry to prenatal care with the participant birth center site. Data are deidentified at the individual level and at the site level. The AABC PDR includes care provided by midwifery‐led birth center practices across all birth settings (birth center, home, and hospital) and includes a variety of health care provider types (midwives [certified professional midwives/licensed midwives, certified nurse‐midwives/certified midwives] and physicians [doctor of osteopathy, family practice, obstetrician‐gynecologist, maternal fetal medicine]). The birth centers included in this study represented a variety of practice models with variations in health care providers’ hospital privileging, ranging from no privileges to privileges in the case of medically indicated transfers of care to full hospital privileges for planned hospital births.

This analysis included data on Medicaid and privately insured clients enrolled in care with the 45 CMMI Strong Start birth centers between 2013 and 2017. Descriptive statistics were used to evaluate the sociodemographic and medical risk factors across 3 groups (privately insured, Strong Start‐enrolled Medicaid beneficiaries, and Medicaid beneficiaries not enrolled in Strong Start). Strong Start enrollees were Medicaid beneficiaries who received midwifery‐led care complemented by peer counselors. Medicaid beneficiaries who did not enroll in Strong Start received midwifery‐led care without peer counselors. Statistical *t* tests were used to evaluate significant variations between the privately insured population and each Medicaid group separately. The significance level was set at *P* < .05. Significant differences between groups during the preliminary analysis informed the risk adjustment model used in the logistic regression.

Sociodemographic variables included years of education, maternal age, gravidity, parity, marital status, and race and ethnicity (self‐attributed by the consumers). Core maternal clinical outcomes included use of medical induction of labor, preterm birth, mode of birth, postpartum hemorrhage, and exclusivity of breastfeeding on discharge. Core neonatal clinical outcomes included low birthweight (<5.5 pounds), 5‐minute Apgar score less than 7, and neonatal intensive care unit (NICU) admission. Transfers of care from birth centers to hospitals based on intention to treat were tracked by noting each client's preference for place of birth at the first prenatal visit, their location of initial admission in labor, and the place of birth.

Logistic regression was used to compare Strong Start Medicaid and non‐Strong Start Medicaid beneficiaries with privately insured consumers to evaluate if poverty was a driver of poor clinical outcomes in this sample. The main outcomes considered in the logistic regression included binary indicators for cesarean birth, induction of labor, epidural analgesia, breastfeeding at discharge, low Apgar score at 5 minutes, low birthweight, and any transfers (antepartum, intrapartum postpartum). Within the logistic regression model, individuals were included in a control group to adjust for medical risk factors (hypertension, diabetes, prior preeclampsia, smoking, substance use disorder, multiparity, and body mass index category [underweight, overweight, obese]). Maternal characteristics were adjusted for based on *t* test analysis between groups. The social adjustment factors included maternal age, educational level (less than college, college, higher than college), race and ethnicity (Black and Latinx), and geographic location (census region Midwest, South, and West). Robust SEs were clustered at the birth center level. All data were analyzed using Stata 16. The AABC PDR is designated as exempt by the New England Institutional Review Board.

## RESULTS

Between 2013 and 2017, 26,259 childbearing people gave birth during the study time frame and met inclusion criteria (privately insured, Strong Start Medicaid enrolled, and non‐Strong Start Medicaid enrolled). Privately insured people represented 57.8% (n = 15,187) of the total sample. The total Medicaid sample was 11,072 (42.2%) and was further divided into 2 groups. Strong Start Medicaid beneficiaries (n = 5793, 52.3%) received midwifery‐led care with enhanced peer counselor services within the birth center model of care, and 5279 (47.6%) were non‐Strong Start Medicaid beneficiaries who received midwifery‐led birth center model care outside of the Strong Start program.

Descriptive statistics for the sample are reported in Table 1. The mean level of education was higher for privately insured consumers and lower for Strong Start and Medicaid beneficiaries, respectively (15.7, 13.0, and 13.3). Privately insured consumers were older on average, compared with Strong Start enrollees and Medicaid beneficiaries (30.2, 26.8, and 27.1), had lower average parity (1.0, 1.2, and 1.3), and were more likely to be from a non‐minoritized group (15.2, 40.9, and 43.8).

**Table 1:** Differences in Sociodemographic and Medical Characteristics Across Payer Types Within American Association of Birth Centers Perinatal Data Registry 2013‐2017, N = 26,259

**Characteristics**	**Total (N = 26,259)**	**Private (n = 15,187)**	**Strong Start** **Medicaid** **(n =5793)**	**Private vs Strong Start** ***P* Value**	**Medicaid (n =5279)**	**Private vs Medicaid** ***P* Value**
**Demographic**						
Years of education, mean (SD)	14.648 (2.764)	15.741 (2.502)	13.007 (2.372)	<.001	13.272 (2.356)	<.001
Maternal age, mean (SD)	28.806 (5.236)	30.173 (4.583)	26.811 (5.411)	<.001	27.063 (5.570)	<.001
Gravidity, mean (SD)	2.499 (1.646)	2.330 (1.499)	2.700 (1.727)	<.001	2.762 (1.881)	<.001
Parity, mean (SD)	1.075 (1.301)	0.955 (1.182)	1.228 (1.391)	<.001	1.255 (1.476)	<.001
Married, n (%)	18,565 (70.7)	13,425 (88.4)	2699 (46.6)	<.001	2433 (46.1)	<.001
White non‐Hispanic, n (%)	19,274 (73.4)	12,878 (84.8)	3423 (59.1)	<.001	2966 (56.2)	<.001
Hispanic, n (%)	3597 (13.7)	956 (6.3)	1257 (21.7)	<.001	1388 (26.3)	<.001
Black, n (%)	1785 (6.8)	577 (3.8)	713 (12.3)	<.001	491 (9.3)	<.001
**Maternal outcomes, n (%)** **^1^**	**23,449**	**13,547**	**5127**		**4783**	
Induction of labor	4179 (17.9)	2425 (17.9)	820 (16.0)	<.001	947 (19.8)	.003
Epidural analgesia	5206 (22.2)	2628 (19.4)	1215 (23.7)	<.001	1444 (30.2)	<.001
Cesarean	2602 (11.1)	1422 (10.5)	646 (12.6)	<.001	5360 (11.2)	.170
Episiotomy	586 (2.5)	325 (2.4)	169 (3.3)	<.001	67 (1.4)	<.001
Postpartum hemorrhage	1290 (5.5)	840 (6.2)	215 (4.2)	<.001	230 (4.8)	<.001
Exclusive breastfeeding	21,432 (91.4)	12,991 (95.9)	4450 (86.8)	<.001	4013 (83.9)	<.001
**Infant outcomes, n (%)** **^1^**	**23,449**	**13,547**	**5127**		**4783**	
Birthweight <5.5lbs	586 (2.5)	271 (2.0)	174 (3.4)	<.001	148 (3.1)	<.001
Apgar score <7 at 5 min	328 (1.4)	163 (1.2)	82 (1.6)	.097	67 (1.4)	.480
NICU admission	258 (1.1)	176 (1.3)	41 (0.8)	.002	38 (0.8)	.003
**Transfers, n (%)**	**26,259**	**15,187**	**5793**		**5279**	
Antepartum	2810 (10.7)	1640 (10.8)	666 (11.5)	<.001	496 (9.4)	<.001
Intrapartum^1^	3330 (14.2)	2100 (15.5)	667 (13.0)	<.001	564 (11.8)	<.001
Newborn^1^	445 (1.9)	284 (2.1)	72 (1.4)	<.001	91 (1.9)	<.001
Postpartum^1^	445 (1.9)	298 (2.2)	62 (1.2)	<.001	86 (1.8)	<.001
**Birth place, n (%)**						
Intended: birth center	19,615 (74.7)	12,407 (81.7)	3881 (67.0)	.006	3294 (62.4)	<.001
Admission place: birth center^1^	13,718 (58.5)	8833 (65.2)	2579 (50.3)	<.001	2305 (48.2)	<.001
Birth place: birth center^1^	13,130 (50.0)	7518 (55.5)	2245 (43.8)	<.001	1965 (41.1)	<.001

^1^ Excludes antepartum transfer (All sample, n = 23,449; private, n = 13,547; Strong Start Medicaid, 5127; Medicaid only, 4783).

Source: American Association of Birth Centers Perinatal Data Registry Data 2013‐2027.

**Table 2:** Adjusted Odds of Adverse Perinatal Birth Outcomes Between Strong Start Medicaid and Non‐Strong Start Medicaid Beneficiaries, Compared with Privately Insured Childbearing People, American Association of Birth Centers Perinatal Data Registry 2013‐2017

	**Private (n = 15,187)**	**Strong Start Medicaid (n = 5793)**		**Medicaid (n = 5279)**	
**Indicators**		**OR (95% CI)**	**aOR (95% CI)**	**OR (95% CI)**	**aOR (95% CI)**
Cesarean	[Reference]	1.150 (0.854‐1.547)	1.155 (0.870‐1.534)	1.074 (0.904‐1.277)	1.061 (0.945‐1.191)
Induction of labor	[Reference]	0.773 (0.509‐1.173)	0.826 (0.605‐1.130)	1.129 (0.846‐1.507)	0.967 (0.829‐1.128)
Epidural analgesia	[Reference]	0.721 (0.385‐1.351)	0.767 (0.520‐1.131)	1.789 (1.144‐2.795)	1.295 (1.096‐1.531)
Breastfeeding	[Reference]	1.257 (0.414‐3.821)	1.109 (0.481‐2.556)	0.226 (0.107‐0.475)	0.570 (0.399‐0.813)
Apgar score <7	[Reference]	1.156 (0.768‐1.740)	1.250 (0.857‐1.823)	1.128 (0.822‐1.546)	0.903 (0.605‐1.348)
Weight <5.5 lb	[Reference]	1.098 (0.653‐1.846)	1.119 (0.765‐1.636)	1.576 (1.196‐2.078)	1.119 (0.903‐1.386)
Antepartum transfer	[Reference]	1.252 (0.854‐1.836)	1.527 (0.971‐2.401)	0.853 (0.645‐1.127)	0.821 (0.643‐1.048)

Abbreviations: aOR, adjusted odds ratio; OR, odds ratio.

Notes: OR reports uncontrolled logistic regression results. The aOR results included control group for medical risk factors (hypertension, diabetes, prior preeclampsia, smoking, substance abuse, multiparity, and body mass index category [underweight, overweight, obese]). The aOR adjusts for maternal characteristics that demonstrated statistical significance using t test analysis: maternal age, education (missing, less than college, college, higher than college), race/ethnicity (Black and Hispanic), and geographic location (census regions Midwest, South, and West). Robust standard errors clustered at the birth center level.

Source: American Association of Birth Centers Perinatal Data Registry Data 2013‐2017.

Although exceeding national benchmarks across all groups, mean maternal outcomes differed between groups prior to adjustment for medical risk factors. Privately insured consumers had similar rates of cesarean birth to the Medicaid population, whereas the Strong Start Medicaid population had the highest rate of cesarean birth (11.0, 11.0, and 13.0). Epidural analgesia use varied between groups, with privately insured consumers maintaining the lowest rates compared with Strong Start and Medicaid, respectively (19.0, 24.0, and 30.2). Privately insured consumers had an 18.0% labor induction rate compared with those with Strong Start and Medicaid (16.0 and 19.8). Postpartum hemorrhage varied between private, Strong Start, and Medicaid (6.2, 4.2, and 4.8). Exclusive breastfeeding on discharge varied between private, Strong Start, and Medicaid beneficiaries (96.0, 86.8, and 84.0).

Average newborn outcomes between payer groups also varied within this sample but exceeded national benchmarks across all payer groups. Compared with privately insured childbearing people, Strong Start and Medicaid beneficiaries had a higher prevalence of low birth weight, on average (2.5, 3.4, and 3.1). Privately insured consumers had higher NICU admissions compared with Strong Start and Medicaid beneficiaries (1.3, 0.8, and 0.8).

Attrition throughout the perinatal episode of care (antepartum, intrapartum, and postpartum) was tracked and also varied by group. Compared with privately insured consumers, the Strong Start group had the highest proportion of the sample lost to antenatal attrition, whereas Medicaid had lower attrition (10.8, 11.5, and 9.4). The privately insured group had the highest proportion of intrapartum attrition compared with Strong Start and Medicaid (15.5, 13.0 and 11.8). Compared with privately insured people, Strong Start beneficiaries had the lowest proportion of newborns transferred (2.1, 1.4, and 1.9) and the lowest proportion of postpartum transfers (2.2, 1.2, and 1.8). Variations in intrapartum, newborn, and postpartum transfer rates between groups may relate to variation in planned place of birth and place of admission. Compared with privately insured consumers, a smaller share of Strong Start and Medicaid beneficiaries intended to give birth at the birth center (81.7, 67.0, and 62.4) and a smaller proportion of clients admitted to the birth center in labor (65.2, 50.3, and 48.2). Compared with privately insured people, Strong Start and Medicaid beneficiaries had a higher proportion of the sample giving birth in the hospital by choice or for medical indications (43.0, 51.0, and 57.0).

Based on these average sociodemographic characteristics and clinical outcomes, logistic regression was used to test whether poverty was an independent driver of maternal and newborn outcomes in this sample (Table 2). Medicaid was used as proxy for poverty, and the sample was analyzed by comparing each of the Medicaid groups with privately insured consumers as the reference point. After controlling for medical risk factors, Strong Start Medicaid beneficiaries achieved similar outcomes to privately insured consumers with no significant differences in maternal or newborn outcomes between groups. Perinatal outcomes included induction of labor (adjusted odds ratio [aOR], 0.86; 95% CI, 0.61‐1.13), epidural analgesia use (aOR, 1.00; 95% CI, 0.68‐1.48), cesarean birth (aOR, 1.16; 95% CI, 0.87‐1.53), exclusive breastfeeding on discharge (aOR, 1.11; 95% CI, 0.48‐2.56), low Apgar score at 5 minutes (aOR, 1.23; 95% CI, 0.86‐1.83), low birth weight (aOR, 1.12; 95% CI, 0.77‐1.64), and antepartum transfer of care after the first prenatal appointment (aOR, 1.53; 95% CI, 0.97‐2.40). Medicaid beneficiaries who were not enrolled in the Strong Start midwifery‐led, peer counselor program demonstrated similar results except for having higher epidural analgesia use (aOR, 1.30; 95% CI, 1.10‐1.53) and significantly lower exclusive breastfeeding on discharge (aOR, 0.57; 95% CI, 0.40‐0.81) than their privately insured counterparts.

## DISCUSSION

In this study, national quality benchmarks were achieved across 45 AABC Strong Start birth centers for both privately and publicly insured populations. Both Medicaid groups included more birthing people who were living at the intersections of poverty, including unmarried, Black, and Hispanic people, with fewer years of formal education than the comparison group of privately insured birth center consumers. The birth center model demonstrated equitable outcomes between private and public payers, suggesting a potential role for the wellness‐based model in the mitigation of poverty‐related health disparities.

Previous studies of birth center care have been limited by homogeneity of the sample and underrepresentation from populations living in poverty.^13^, ^14^ Additionally, studies demonstrating favorable outcomes at birth centers compared with hospitals have been critiqued as skewed toward a group of people who self‐selected into birth center care and who do not represent a generalizable subset of birthing people.^15^ However, midwifery‐led birth center care has previously been shown to be desirable for a large range of birthing people with a variety of sociodemographic characteristics, suggesting that lack of use of the model relates to lack of access, not lack of preference.^16^, ^17^, ^18^, ^19^, ^20^

This analysis demonstrated that the beneficial outcomes from birth center‐led prenatal care persist between payer groups and across sociodemographic categories for most of the variables evaluated. The Medicaid Strong Start group was able to achieve equitable outcomes compared with privately insured people on all measures analyzed, including socially mediated indicators and those requiring social support (epidural analgesia and exclusive breastfeeding). The combination of midwifery‐led birth center care and peer counselors has a role in mitigation of societal risks and their impact on childbearing families.

The results of this research further illustrate the potential impact of evidence based psychosocial support interventions, specifically peer counselors within a birth center model.^21^, ^22^, ^23^, ^24^ This finding furthers the science on midwifery‐led care, including the enhanced care components of community health workers, doulas, lactation consultants, and longer prenatal visits. Questions for future research include whether the racial concordance of the health care providers and peer counselors had a dose‐dependent effect on mitigating or narrowing disparities.^25^, ^26^, ^27^

Limitations to sustainability, scale, and spread of the birth center model have been noted in previous research, including lack of payment for enhanced care services, like peer counselors, doulas, and community health workers.^8^ Although states are required to provide Medicaid coverage for pregnant people, there is wide variation in covered services across states related to midwifery‐led care.^8^ In the Strong Start impact analysis, childbearing people with high social needs who received care at birth centers had better outcomes despite the US health care financing system not having been demonstrated to reliably pay for the core components of birth center care (eg, sustainable midwifery care provider fees, facility fees, and enhanced care services including longer prenatal visits with midwives and the services of community health workers, doulas, lactation consultants, and health education).^1^, ^8^

The findings of this study support the call to expand access to birth center care as part of a national initiative to improve perinatal outcomes. The optimal outcomes of vaginal birth, spontaneous labor, and healthy term, normal weight newborns transcended payer status in this sample, suggesting that outcomes of midwifery‐led birth centers are robust at the population health level. Medicaid programs can achieve improved outcomes by aligning their policies with the current evidence on effective interventions for optimal outcomes among Medicaid participants. The processes of enhanced care service (eg, doulas, breastfeeding peer counselors, community health workers, and lactation consultants, as well as longer prenatal visits with midwives) require sustainable reimbursement by Medicaid.^8^ Addressing known barriers that prevent the spread and scale of the model within minoritized communities is a high‐impact policy strategy.^8^

More research is needed to understand the high leverage components of the innovative Strong Start birth center model. Other variables of interest relate to the business structure of the birth center, for‐profit or not‐for‐profit, and whether there is a relationship to outcomes.^28^, ^29^

The organization of the birth center practices within this sample vary, including whether midwives have privileges at a hospital and whether the practice offers midwifery‐led hospital birth. It was outside the scope of this research to explore these variables. More research is needed to understand these relationships, to further inform policy and move the model to scale in the United States. In this study, compared with privately insured birthing people, those in both Medicaid groups were significantly more likely to be admitted to the hospital in labor. Decisions regarding place of birth admissions were related to the birthing persons preferences, as well as medical risk factors in this sample.^30^ As the model is brought to scale in the United States, the organization of practice model should be driven by community needs and preferences, one of the AABC Standards for Birth Centers.^4^ This study supports the growth of midwifery‐led practice models that offer birth center, hospital admission, or both if preferred or warranted.

A notable limitation of this study is the lack of generalizability due to unquantifiable selection bias within the population and unique characteristics of the 45 Strong Start birth centers. There is an assumed selection bias of consumers who choose birth center care, although, to date, no research has been published quantifying the existence or impact of selection bias upon initiation of prenatal care within the birth center model. The Strong Start birth centers have been recognized for their culture of quality with a high percentage of sites accredited by the Commission for Accreditation of Birth Centers (CABC). These results are not generalizable in settings that do not follow AABC Standards and are not accredited by the CABC.^4^

## CONCLUSION

The optimal outcomes achieved through birth center care were not limited to clients with private insurance; they extend to those living at the intersections of poverty. The increased risk for poor outcomes in Medicaid populations can be mediated by midwifery‐led care including enhanced care services with peer counselors. Birth center model care achieves care that is safe and above national quality standards for both public and private payer patients.

## CONFLICT OF INTEREST

The authors have no conflicts of interest to disclose.
